# Targeting Tissular Immune Response Improves Diagnostic Performance of Anti-*Saccharomyces cerevisiae* Antibodies (ASCA) in Crohn’s Disease

**DOI:** 10.1371/journal.pone.0080433

**Published:** 2013-11-26

**Authors:** Daniel Bertin, Jean-Charles Grimaud, Nathalie Lesavre, Chahine Benelmouloud, Ariadne Desjeux, Stéphane Garcia, Sophie Desplat-Jégo

**Affiliations:** 1 Service d’Immunologie, Pôle de Biologie, Hôpital de la Conception, Assistance Publique-Hôpitaux de Marseille, Marseille, France; 2 Aix-Marseille Université, Centre national de la recherche scientifique, Neurobiologie des Interactions Cellulaires et Neurophysiopathologie UMR 7259, Marseille, France; 3 Service de Gastroentérologie, Hôpital Nord, Assistance Publique-Hôpitaux de Marseille, Marseille, France; 4 Centre d’Investigations Cliniques, Hôpital Nord, Assistance Publique-Hôpitaux de Marseille, Marseille, France; 5 Laboratoire d’Anatomie Pathologique, Hôpital Nord, Assistance Publique-Hôpitaux de Marseille, Marseille, France; Centro di Riferimento Oncologico, IRCCS National Cancer Institute, Italy

## Abstract

Antibodies against *Saccharomyces cerevisiae* (ASCA) and *Escherichia coli* outer membrane porin C (anti-OmpC) are known to be detectable in the serum of patients with Crohn’s disease (CD) but display a very poor sensitivity for the disease especially in forms with isolated colonic involvement. In this study we aimed at **e**valuating performances of these markers in supernatant of cultured colonic biopsies.

Patients with colonic CD (n =  67), ulcerative colitis (UC) (n = 35) and control individuals (n = 37) were prospectively recruited for colonoscopy pinch biopsies and blood sampling. Serum and supernatant of culture tissues were analyzed for ASCA and anti-OmpC. Direct immunofluorescence was also performed on colonic tissues for total IgA detection.

We detected for the first time ASCA IgA/IgG and anti-OmpC IgA in cultured colonic tissue supernatants. For both markers, sensitivities for diagnosing CD were better in supernatants (ASCA: 53.7%, anti-OmpC: 28.4%) than in serum (ASCA: 31.3%, anti-OmpC: 22.4%). Combination of results from a panel of these tests gave the greatest sensitivity ever described for CD diagnosis in colonic forms (70.2%).

In this study, we described, for the first time, ASCA in supernatant of colonic tissue cultures. This assaying approach in CD diagnosis should be taken into consideration in the future especially in CD forms with isolated colonic involvement.

## Introduction

Inflammatory bowel diseases (IBD) such as Crohn’s disease (CD) or ulcerative colitis (UC) are heterogeneous chronic intestinal inflammatory disorders occurring in genetically predisposed individuals in association with a host immune response against gut flora. In most cases, a diagnosis of CD or UC can be made with high certainty but sometimes and especially in case of exclusive colonic localization of the disease, the diagnosis is difficult. Several serum antibodies against microbial antigens have been proposed as serological markers for CD diagnosis [1]. Among them, anti-*Saccharomyces cerevisiae* antibodies (ASCA) and anti-OmpC antibodies are directed against phosphopeptidomannan of the cell wall of the yeast [2] and outer membrane porin C of *Escherichia coli,* respectively. ASCA are anti-glycan antibodies that were first described in IBD and that can be predictive for CD development in asymptomatic individuals [3]. ASCA can also be detected in patients with auto-immune diseases such as antiphospholipid syndrome, systemic lupus erythematosus where they cross-react with autoantigens [4] [5]. However in the event of IBD suspicion, ASCA reported high specificity for CD. It is now well established that ASCA could be useful for differentiating CD from UC [6]. But despite good specificity for CD, ASCA and anti-OmpC display too low sensitivities (less than 60%) for CD [6] [7] [8] [9]. In fact, especially in colonic form of CD, the situation in which serological markers should be the most relevant in order to distinguish CD from UC, the sensitivity of both test is less than 40% [10] [11]. So, the diagnostic role of these immunological markers in clinical practice appears to be limited due to this low sensitivity. Data about luminal presence of these antibodies in IBD patients are lacking. Nevertheless it could be of interest and more informative to target the local immune tissue response rather than the blood systemic response in our processes of antibody detection for CD diagnosis. We have chosen to focus our study on colonic form of CD in which serological markers offer the worst performances and for the first time we examined whether: i) ASCA and anti-OmpC can be detected in supernatants of cultured colonic pinch biopsies issued from CD patients, ii) the changing of biological fluid (supernatant versus serum) used for ASCA and anti-OmpC testing could be a simple way to improve the diagnostic role of these antibodies for CD diagnosis.

## Materials and Methods

### Patients

Consecutive patients suffering from IBD including CD with isolated colonic involvement or UC and control individuals undergoing colonoscopy for functional intestinal disorders without IBD were prospectively recruited for colonoscopy pinch biopsies and peripheral venous blood sampling. All patients were followed up at the gastroenterology unit of the University Hospital in Marseille, France. The diagnosis was based on clinical, radiological, endoscopic examination and histological findings using previously described criteria [12] [13]. The disease activity of CD patients was evaluated by calculating the Crohn Disease Activity Index [14]. This study has been approved by the local ethic committee Comité de Protection des Personnes (CPP) Sud Méditerranée V. All patients gave their written informed consent. The clinical trial protocol received the clinical.gov number NCT00769236. Clinical features of the diseases were collected from medical charts in clinical research forms. After serum separation, blood samples were stored at –80°C until further analysis.

### Colonic biopsies cultures

During total colonoscopy under general anaesthesia, eight biopsies were performed in each part of the right, transverse and left colon. Samples were i) fixed in 4% buffered formalin for histological examination, ii) put in RPMI medium for culture technique or iii) deep frozen in liquid nitrogen then stored at –80°C for immunofluorescence technique.

For histological examination, biopsies were fixed in 4% buffered formalin and paraffin embedded. Four thick sections were performed and stained with hematoxylin and eosin for routine examination. Inflammation was assessed using the following morphological criteria: none, non-specific chronic (predominance lymphocytes and/or plasma cells), non-specific acute (predominance of polymorphonuclear cells), specific granulomatous.

After biopsy, colonic tissues put in RPMI medium on bedside were immediately transported at room temperature to the immunology laboratory. Then, they were washed 3 times with phosphate buffered saline (PBS) and transferred into 24-well culture plates (Nunc®, Naperville, IL, USA) with RPMI 1640 medium supplemented with 10% fetal bovine serum and antibiotics (penicillin 100 U/ml, streptomycin 100 µg/ml). The plates were incubated for 72 hours at 37 °C in atmosphere with 5% CO2. Then supernatants were collected, centrifuged, aliquoted and stored at –80°C until analysis.

### ASCA IgA /IgG detection

ASCA IgA and IgG were detected by indirect immuno-fluorescence (IIF), in a blinded way, using a commercially available detection kit (Euroimmun, Germany). Sera were diluted in phosphate buffer (1∶500 for IgG and 1∶50 for IgA) and supernatants were tested without dilution. Samples were incubated for 30 min on slides with smears of *Saccharomyces cerevisiae*. After a washing step, fluorescein-conjugated goat anti-human IgG or IgA was added. After three more washes, mounted slides were observed with epifluorescence microscope (Leica DM1000®, Leica Microsystems, Germany) by two experienced readers.

### Anti-OmpC IgA detection

IgA antibodies against bacterial OmpC were detected, in a blinded way, with the commercially available ELISA test QUANTA Lite OMP PLUS*®* (INOVA Diagnostics, San Diego, CA, USA) according to the manufacturer’s protocol. Serum results are expressed in arbitrary units (A.U) with a cutoff for positivity of 25 A.U. Due to the lack of a manufacturer’s cutoff for supernatants we had to calculate it. We defined the cutoff for supernatants positivity as the 97^th^ centile of the optic density (O.D) obtained for the UC + control group. This cut off stands at 0.831 absorbance units. Reproducibility for anti-OmpC IgA detection in supernatants was calculated and the inter-assay variation coefficient reached 10.2%.

### IgA detection on colonic tissues

IgA detection was carried out on 5-mm thick frozen sections (cryostat Leica CM 3050, Rueil Malmaison, France) using a direct immunofluorescence technique. The procedure was realized using an FITC conjugated F(ab')2 recognizing human IgA (Biorad, Marnes-la-Coquette, France) diluted at 1:40 and incubated 30 min at room temperature. IgA deposits were assessed and described as linear or granular and located in epithelial cells or in stromal compartment.

### Statistical analysis

Sensitivity was measured as the probability of a positive result in a patient with CD; specificity was measured as the probability of a negative result in a patient with UC or non IBD intestinal disorders (control group). The positive predictive value (PPV) was the probability of having CD and a positive result while the negative predictive value (NPV) was the probability of having UC or non IBD intestinal disorders and negative result. Comparison of different groups was done using the Chi-square test or Fisher's exact test when appropriate. The agreement between ASCA and anti-OmpC detection in both sera and supernatants was evaluated using Cohen's Kappa coefficient [15] which takes on the value i) zero if there is no more agreement between two tests as can be expected on the basis of chance, ii) 1 if there is perfect agreement. It is considered that Kappa values lower than 0.4 represent poor agreement, values between 0.4 and 0.80 moderate to substantial agreement, and values higher than 0.80 almost perfect agreements. Negative Kappa indicates the level of agreement was below that expected by chance. Data were analyzed using Graphpad Prism 5 (Graphpad software, San Diego, CA, USA) and the threshold for statistical significance was set at p<0.05.

## Results

### Patient population

We prospectively and consecutively included a total of 139 patients: 67 CD patients with isolated colonic involvement, 35 patients with UC and 37 control individuals undergoing colonoscopy for functional intestinal disorders without IBD. The median age at study was 43.4 [19–84] for CD group, 42.9 [18–74] for UC group and 58.1 [22–79] for the control group. The sex ratios (M/F) were respectively 24/43, 20/15 and 17/20 for CD, UC and control group.

Among CD patients, the diagnosis of CD was set under the age of 20 years for 13 patients (19.4%) and beyond the age of 20 years for 54 patients (80.6%) and so the duration of disease was superior at 5 years for 60 patients (89.6%). The disease was active for 12 patients (17.9%) and quiescent for 55 patients (82.1%) at the time of the study. A minority of CD patients smoked (46.3%) and 36.5% had yet suffered from extra intestinal symptoms of the disease.

At the time of study, a macroscopic inflammation at colonoscopy was detected in 28 patients (41.8%). Histological findings available in only 35 CD patients showed epithelioïd granuloma in 11% of patients.

### Prevalence of ASCA and anti-OmpC in patient serum

**Figure 1:**
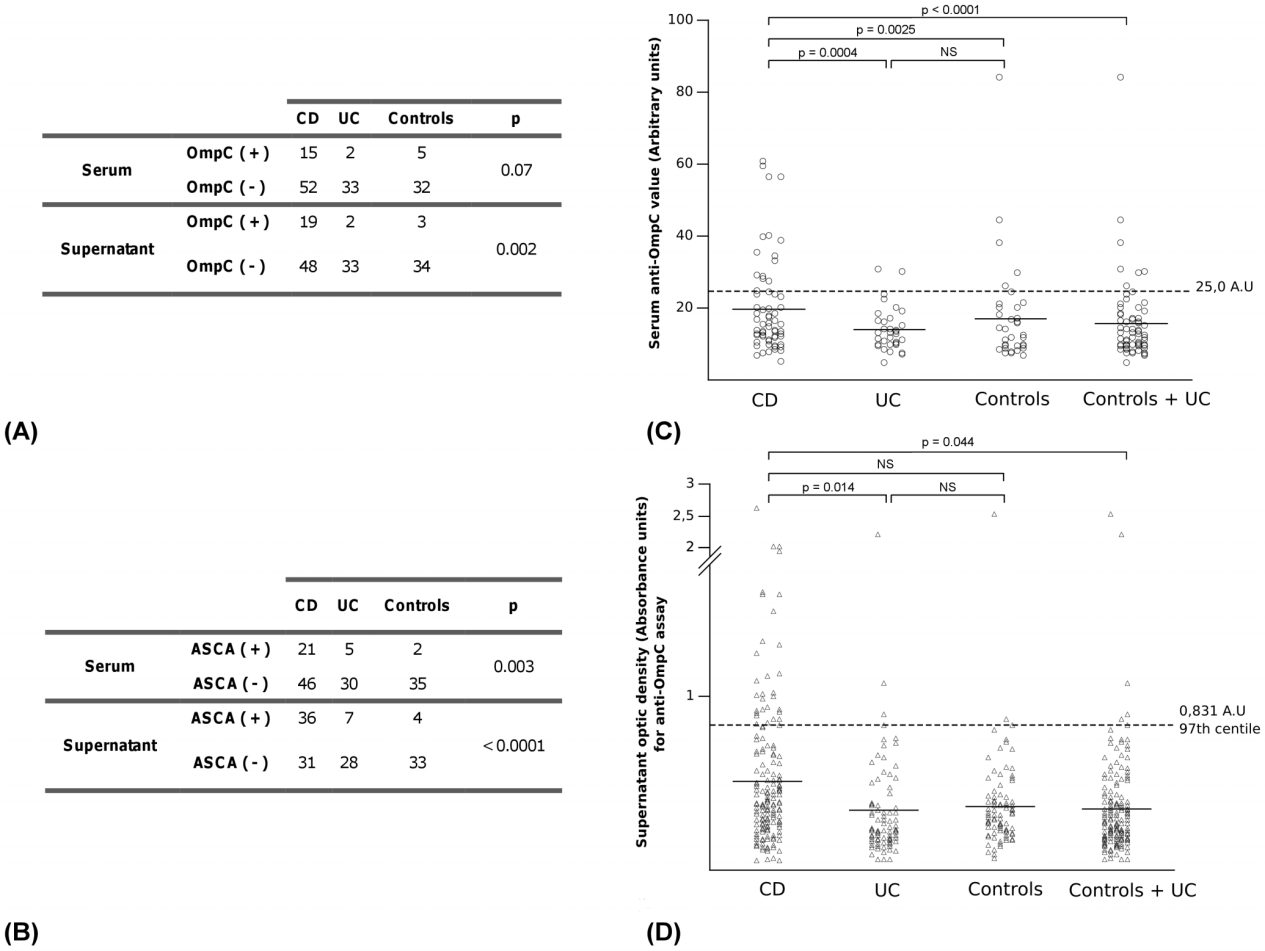
ASCA and anti-OmpC detection in sera and supernatants for each clinical group. (A) Qualitative results of anti-*Saccharomyces cerevisiae* antibodies (ASCA) test by IFI in sera and supernatants for each clinical group. One patient is considered as ASCA positive in case of positivity of ASCA IgA and/or ASCA IgG. (B) Anti-outer membrane porine C antibodies (anti-OmpC) detection by ELISA in sera and supernatants for each clinical group.(C) Distribution of anti-OmpC antibody levels in patient sera. According to manufacturer recommendations, anti-OmpC antibody levels obtained with ELISA are considered as significant when superior or equal to 25 arbitrary units (A.U). (D) Distribution of optical densities (O.D) obtained with anti-OmpC ELISA in patient supernatants issued from culture of colonic tissues. The threshold of positivity (0.831 absorbance units) is the 97th centile of the O.D obtained for the controls + UC group. NS: no statistically significant difference. The comparison between different groups is done using the Student's t-test with p = 0.05 as significance level. CD: Crohn’s disease, UC: ulcerative colitis, Controls: patients suffering from functionnal intestinal disorders but not from CD or UC.

Serum ASCA (IgA and/or IgG) but not anti-OmpC were statistically more prevalent (p = 0.003) in CD group than in UC + control group (Fig 1A-1B). Moreover, anti-OmpC titers were significantly higher in CD group than in UC + control group (p<0.0001) (Fig 1C). In our population (colonic form), sensitivity and specificity of serum ASCA for CD were respectively 31.3% and 90.3% while for anti-OmpC (IgA) we found sensitivity at 22.4% and specificity at 90.3% for CD (Table 1).

**Table 1:** Performances of ASCA and anti-OmpC tests.

	Se (%)	Sp (%)	PPV (%)	NPV (%)
Serum ASCA	31.3	90.3	75	58.6
Serum OmpC	22.4	90.3	68.2	55.6
Supernatant ASCA	53.7	84.7	76.6	66.3
Supernatant OmpC	28.4	93.1	79.2	58.3
**Test combination:**				
Serum ASCA	59.7	79.8	72.7	67.9
and supernatant ASCA				
Serum OmpC	46.3	86.1	75.6	63.3
and supernatant OmpC				
Serum ASCA	41.8	82	68.3	60.2
and serum OmpC				
Supernatant ASCA	64.2	79.2	74.1	70.4
and supernatant OmpC				
Serum ASCA	70.2	69.4	68.1	71.4
and supernatant ASCA				
and serum OmpC				
and supernatant OmpC				

ASCA: Anti-*Saccharomyces cerevisiae* antibodies, anti-OmpC: anti-outer membrane porine C antibodies, Se: sensitivity, Sp: specificity, PPV: positive predictive value, NPV: negative predictive value all expressed in percents.

### Prevalence of ASCA and anti-OmpC in colonic culture supernatants

We showed for the first time that ASCA (IgA and/or IgG) and anti-OmpC (IgA) were detectable in colonic tissue culture supernatants. We found ASCA in 36/67 CD supernatants and anti-OmpC in 19/67 (Fig 1A-1B). ASCA and anti-OmpC were statistically more prevalent (p<0.0001 and p = 0.002 respectively) in CD group than in UC + control group. Among 36 CD patients with ASCA in supernatant, we found that 15 of them (41.7%) displayed both ASCA IgA and IgG, 14 patients (38.9%) only displayed ASCA IgA and 7 patients (19.4%) only displayed ASCA IgG.

### Comparative performances of ASCA and anti-OmpC for CD diagnosis in serum versus culture supernatants

**Figure 2:**
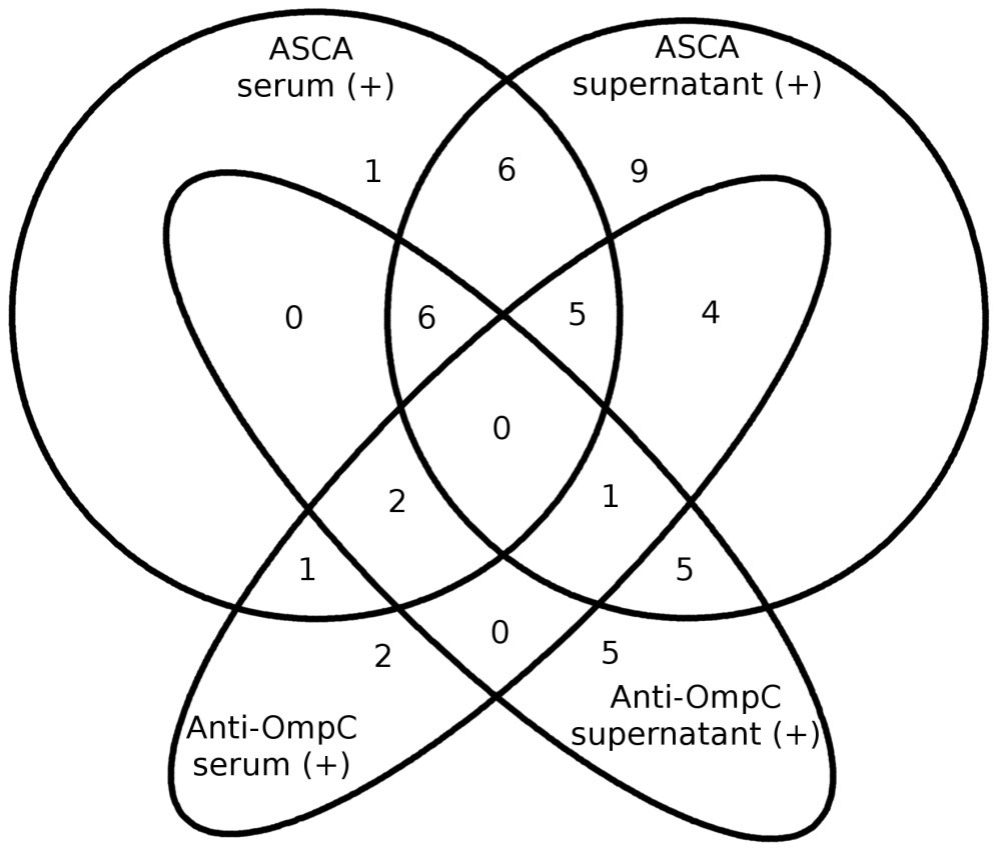
Profile of ASCA and anti-OmpC antibodies in sera/supernatants of the 47 CD patients displaying at least one positive test. ASCA: anti-*Saccharomyces cerevisiae* antibodies, anti-OmpC: anti-outer membrane porine C antibodies.

Nineteen ASCA seronegative CD patients were positive for ASCA in culture supernatant. Among ASCA negative CD patients, 7 were anti-OmpC positive in serum (2 patients) or in culture supernatant (5 patients) (Figure 2). We have not found any patient simultaneously positive for the four tests (Figure 2). Among 67 patients with CD, 20 patients remained negative for ASCA and anti-OmpC whatever the analyzed sample (serum or supernatants) (Figure 2)

ASCA sensitivity for CD diagnosis was higher with supernatants (53.7%) than with serum (31.3%). This increase of sensitivity offered by supernatant analysis has also been observed for anti-OmpC even though it was a very moderate increase (28.4% versus 22. 4%) (Table 1). By differently combining the results from the four different possible tests and by considering positivity of at least one test, we observed an increase of sensitivity for CD diagnosis (Table 1). The combination of ASCA analysis in both serum and supernatants gave a sensitivity of 59.7%. In the same way, associating the four possible tests allowed to reach sensitivity for CD diagnosis of 70.2% (Table 1).

### Study of relationship between IgA intestinal deposit and ASCA/anti-OmpC antibody detection in supernatants

Biopsies issued from 61 patients (twenty nine CD patients and 32 patients from UC + control group ie more than 150 biopsies) were analyzed by direct immunofluorescence for studying total IgA colonic deposits. We visualized granular IgA deposits in colonic mucosa both in epithelium and in stroma in all clinical groups. In fact, we observed no significant relationship between IgA colonic deposits and i) the diagnosis of CD or ii) ASCA/anti-OmpC antibody detection in supernatants (Table 2). In fact we could detect IgA deposits on control patients and we did not find any staining in several CD patients even though they were positive for ASCA IgA in culture supernatants.

**Table 2:** Study of IgA intestinal deposit and ASCA/anti-OmpC antibody detection in supernatants for each clinical group.

			IgA colonic		Kappa
			deposit by DIF		coefficient
			+	–	
	IgA ASCA supernatants	+	15	2	–0.13
**CD**		–	12	0	
**(n = 29)**	IgA anti-OmpC supernatants	+	7	0	0.05
		–	20	2	
**Controls**	IgA ASCA supernatants	+	4	0	0
**+**		–	28	0	
**UC**	IgA anti-OmpC supernatants	+	2	0	0
**(n = 32)**		–	30	0	

IgA intestinal deposit was evaluated by direct immunofluorescence (DIF). The agreement between IgA intestinal deposit and ASCA / anti-OmpC antibody detection in supernatants of each clinical group was evaluated using Cohen's kappa coefficient. ASCA: Anti-*Saccharomyces cerevisiae* antibodies, anti-OmpC: anti-outer membrane porine C antibodies, CD: Crohn’s disease, UC: ulcerative colitis, Controls: patients suffering from functional intestinal disorders but not from CD or UC.

### Study of correlation between ASCA or anti-OmpC detection and CD patient features

As showed in table 3, serum ASCA antibodies were more frequent in men than in women (p  =  0.014). Nevertheless, we did not find any correlation between ASCA positivity in serum and/or supernatant and the other studied clinical features (age at sampling, smoking, disease duration, macroscopic inflammation at colonoscopy, epithelium granuloma, extra intestinal symptoms and treatment). In our CD cohort, we did not find any correlation between anti-OmpC positivity and the studied clinical features (Table 4).

**Table 3:** Study of correlation between ASCA status and CD clinical features.

Clinical features of CD	Number	Serum	*p*	Supernatant	*p*
	of patients (%)	ASCA + (%)		ASCA + (%)	
**Gender**					
Men	24 (35.8)	12 (50)	*0.014*	15 (63)	*NS*
Women	43 (64.2)	9 (21)		21 (49)	
**Age at sampling**					
> 40 yrs	38 (56.7)	12 (32)	*NS*	23 (61)	*NS*
< 40 yrs	29 (43.3)	9 (31)		13 (45)	
**Disease activity**					
Active	12 (17.9)	6 (50)	*NS*	6 (50)	*NS*
Quiescent	55 (82.1)	15 (27)		30 (55)	
**Age at diagnosis**					
> 20 yrs	54 (80.6)	16 (30)	*NS*	29 (54)	*NS*
< 20 yrs	13 (19.4)	5 (38)		7 (54)	
**Duration of disease**					
> 5 yrs	60 (89.6)	20 (33)	*NS*	34 (57)	*NS*
< 5 yrs	7 (10.4)	2 (29)		2 (29)	
**Smoking**					
Yes	31 (46.3)	11 (35)	*NS*	16 (52)	*NS*
No	36 (53.7)	10 (28)		20 (56)	
**Macroscopic Inflammation**					
**at colonoscopy at inclusion**					
Presence	28 (41.8)	12 (43)	*NS*	16 (57)	*NS*
Absence	39 (58.2)	9 (23)		20 (51)	
**Epithelioid granulomas**					
Presence	4 (11.4)	3 (75)	*NS*	3 (75)	*NS*
Absence	31 (88.6)	7 (23)		17 (55)	
**Extra-intestinal symptoms**					
Presence	23 (36.5)	6 (26)	*NS*	10 (43)	*NS*
Absence	40 (63.5)	12 (30)		22 (56)	
**Digestive surgery**					
Yes	35 (52.2)	14 (40)	*NS*	23 (66)	*NS*
No	32 (47.8)	7 (22)		13 (41)	
**Medical treatment**					
Yes	43 (64.2)	13 (30)	*NS*	23 (53)	*NS*
No	24 (35.8)	8 (33)		13 (54)	

ASCA: Anti-*Saccharomyces cerevisiae* antibodies, CD: Crohn disease, *NS*: no statistically significant difference. The comparison of different groups is done using the chi-square test or Fisher's exact test when appropriate with p = 0.05 as significance level.

**Table 4:** Study of correlation between anti-OmpC antibodies status and CD clinical features.

Clinical features of CD	Number	Serum	*p*	Supernatant	*p*
	of patients (%)	OmpC + (%)		OmpC + (%)	
**Gender**					
Men	24 (35.8)	8 (33)	*NS*	9 (38)	*NS*
Women	43 (64.2)	7 (16)		10 (23)	
**Age at sampling**					
> 40 yrs	38 (56.7)	10 (26)	*NS*	12 (32)	*NS*
< 40 yrs	29 (43.3)	5 (17)		7 (24)	
**Disease activity**					
Active	12 (17.9)	4 (33)	*NS*	5 (42)	*NS*
Quiescent	55 (82.1)	11 (20)		14 (25)	
**Age at diagnosis**					
> 20 yrs	54 (80.6)	12 (22)	*NS*	14 (26)	*NS*
< 20 yrs	13 (19.4)	3 (23)		5 (38)	
**Duration of disease**					
> 5 yrs	60 (89.6)	14 (23)	*NS*	17 (28)	*NS*
< 5 yrs	7 (10.4)	1 (14)		2 (29)	
**Smoking**					
Yes	31 (46.3)	5 (16)	*NS*	10 (32)	*NS*
No	36 (53.7)	10 (28)		9 (25)	
**Macroscopic inflammation**					
**at colonoscopy at inclusion**					
Presence	28 (41.8)	9 (32)	*NS*	9 (32)	*NS*
Absence	39 (58.2)	6 (15)		10 (26)	
**Epithelioid granulomas**					
Presence	4 (11.4)	0 (0)	*NS*	2 (50)	*NS*
Absence	31 (88.6)	10 (32)		6 (19)	
**Extra-intestinal symptoms**					
Presence	23 (36.5)	5 (22)	*NS*	6 (26)	*NS*
Absence	40 (63.5)	10 (25)		12 (30)	
**Digestive surgery**					
Yes	35 (52.2)	9 (26)	*NS*	13 (37)	*NS*
No	32 (47.8)	6 (19)		6 (19)	
**Medical treatment**					
Yes	43 (64.2)	9 (21)	*NS*	13 (30)	*NS*
No	24 (35.8)	6 (25)		6 (25)	

anti-OmpC: anti-outer membrane porine C antibodies, CD: Crohn’s disease, *NS*: no statistically significant difference. The comparison of different groups is done using the chi-square test or Fisher's exact test when appropriate with p = 0.05 as significance level.

## Discussion

In the present study, we assessed the clinical value of ASCA and anti-OmpC tests performed in supernatant issued from cultured colonic biopsies for the diagnosis of CD in comparison with their detection in serum. Moreover, to our knowledge this work is the first study focusing on colonic form of CD.

We described here for the first time the detection of ASCA IgA/IgG and anti-OmpC IgA in cultured colonic tissue supernatants. Moreover we found that these markers were significantly more prevalent in CD patient group than in UC + controls group. In our colonic CD population, the correlation study between the presence of these markers and various clinical features has only shown a greater prevalence of serum ASCA in men than in women. For both markers, sensitivities for diagnosing CD were greatly better in supernatants (ASCA: 53.7%, anti-OmpC: 28.4%) than in serum (ASCA: 31.3%, anti-OmpC: 22.4%) especially for ASCA. Combining results from a panel of these tests gave the greatest ASCA sensitivity ever described for CD diagnosis in colonic forms (70.2%).

It is now widely accepted that defects in innate immunity cause a loss of the mucosal tolerance to microbial components [16]. Moreover, in the latter decades, various antibodies against microbial epitopes have been studied in CD and UC but many of these studies concluded that these serological markers were of poor diagnostic interest by a great lack of sensitivity for CD [17]. In fact all these studies were searching markers in the blood only and did not focus on intestinal tissue which is the main place where IBD disorders occur. We suggest here that the poor performances of these markers are related to the lack of consideration for the immune local response occurring in the intestinal tissue. In such context, an interesting approach has been proposed by Carroccio et al [18] who detected anti-endomysial antibodies in cultured duodenal mucosa biopsy from celiac patients. However, to our knowledge, there are no studies that performed mucosal intestinal biopsies to verify if ASCA and anti-OmpC are detectable in culture supernatant. We have showed here that ASCA and anti-OmpC from both isotypes IgA and IgG could be detected from colonic tissue cultures. Nevertheless, the detection of IgA in culture supernatant brings up questions about the origin of these IgA. In fact the presence or not of a secretory piece in the detected IgA remains to be determined since the conjugate we used in routine was not targeted against this component. Indeed secretory IgA containing a secretory piece may be of intestinal origin. A further analysis with an antibody targeting secretory piece should be of interest. Moreover, the IgA assayed in the supernatant may be related to biopsy contamination by blood or mucus but the fact that we have rinsed the tissues several times before IIF test seems to exclude this hypothesis. The origin of this IgA secretion remains uncertain but isolated lymphoid follicles can be found in the colon and it has been shown that their number, diameter and density increase in inflammatory conditions such as IBD [19]. ILF are gut-associated lymphoid tissues that are supposed to play a role in immune surveillance and colonic repair mechanisms [20].These lymphoid follicles as well as Peyer’s patches of the small intestine contains numerous dendritic cells, macrophages, T and B cells and could be a source for the IgA detected in the supernatants of cultured colonic biopsies. Whatever the origin of this IgA secretion, we suggest that it could be a very localized and intermittent phenomenon since i) among 3 biopsies done per patient, there is often only one which gives a positive result, ii) the positive sample was not necessarily the sample recovered from a macroscopically inflammatory area, iii) the amount of produced IgA are low and requires to work with undiluted supernatants. In a recent and interesting study, Tang et al [21] have shown that ASCA could be detected in feces suggesting that ASCA could be excreted. To better investigate the origin of the IgA detected in our supernatants we have performed in parallel direct immunofluorescence on the studied colonic tissue. We found then no difference between control and CD cohort: in both populations, we could detect IgA deposits in colonic mucosa and they were not associated with the presence or not of ASCA in supernatants. Nevertheless we have only studied the total IgA contingent since, for technical reasons, we were not able to distinguish ASCA IgA among total IgA. Then we can’t exclude that ASCA IgA deposits are more frequent during CD than UC.

ASCA and anti-OmpC are serological markers that have been proposed to differentiate CD from UC. Previous studies showed that serum ASCA specificity for CD is almost 95% while sensitivity is low since high titers of ASCA IgG and/or IgA were found in only 50–70% of all CD patients [22] [23] and less than 40% in colonic form of the disease. In our study we have chosen to work only on CD with isolated colonic involvement in order to target the population of CD patients in which serological test could be the most relevant and helpful. So it is not surprising to obtain in our hands a sensitivity of 31.3% for ASCA test and 22.4% for anti-OmpC test in serum. In fact the expression of ASCA in serum is preferentially associated with ileal forms of CD [24] [10] and it has also been observed for anti-OmpC [7]. Two recent meta-analyses [6] [9] indicated that positive ASCA serum status is associated with early-onset age, ileal involvement, complicated behavior, perianal disease and requirement for surgery in CD. In a previous study [25] we agreed with some of these data but in the present study we highlighted the specificities of a CD patient cohort with isolated colonic involvement and no pediatric case have been included. For this particular cohort of CD, to our knowledge, literature data are now lacking. Moreover, only a few studies deal with the clinical interest of anti-OmpC in serum of CD patients. Dubinsky [26] studied a pediatric cohort of 196 patients with CD and showed that the positivity of anti-OmpC in serum was associated with a more severe form of the disease. Papp [8] and Ferrante [7] showed that CD adult patients with anti-OmpC positive had more often surgery than those who were anti-OmpC negative. They also observed that the positivity of anti-OmpC in serum of patients with CD was more frequently associated with long-term evolution of the disease and early age diagnosis. However, we have not made such observations in our CD population for anti-OmpC as well in serum as in supernatants even if we have used the same reagents. One explanation could be that Papp and Ferrante have not distinguished the colonic form of CD from the others in their correlation study with clinical features. We didn’t find any correlation between activity of CD and anti-OmpC detection in serum so we are in accordance with results from a recent study that didn’t support the use of OmpC serology testing in disease activity assessment, or in screening for active CD [27]. Surprisingly, we described for the first time a correlation between gender and the positivity of ASCA in serum. In fact our data showed that men with colonic form of CD displayed more frequently ASCA positivity than women with the same localization of the disease (p =  0.014). This fact has never been described in ileal or anal forms and so remains to be confirmed by other teams.

Antineutrophil cytoplasmic antibodies (ANCA) were also investigated in sera and supernatants but we didn’t detect any ANCA by IIF in supernatants of cultured colonic biopsies (data not shown) thus there is no interest of searching them in culture supernatants.

However, our results showed also that ASCA and anti-OmpC detection in culture supernatant improved the sensitivity of ASCA and anti-OmpC for the detection of CD patients. In our study, we were able to detect 19 patients who were not detected by these assays performed in serum alone: 9 were positive for ASCA, 5 for anti-OmpC and 5 for both tests in supernatants. By using tests in supernatants we were then able to successfully commute the CD biological marker status in 19 out of 67 patients.

We did not reveal a link between the presence of these markers and CD activity at the time of completion of sampling. The lack of relationship between disease activity and the presence of antibodies is in favor of the absence of a direct pathogenic role of these antibodies. It should also be noted that we only measured the IgA anti-OmpC (IgG isotype not commercially available) while ASCA were evaluated for both IgA and IgG isotypes. This difference may explain in part the greater sensitivity of ASCA for CD compared to anti-OmpC. Further studies could evaluate the performance gain of IgG anti-OmpC since the first highlighted anti-OmpC antibody was IgG isotype [28].

We also evaluated the interest of a combination of tests ASCA / anti OmpC performed in the serum and/or in the supernatant. For both ASCA and anti-OmpC, supernatants analysis added to serum result, significantly increases the sensitivity of the tests: ASCA gains 28.4% sensitivity and anti-OmpC gains 23.9% sensitivity. By using a combination Serum ASCA and/or ASCA supernatant and/or OmpC serum the sensitivity amounted to 62.7%, which is slightly more than the 59.7% sensitivity of the combination ASCA serum and/or ASCA supernatant: the value of serum anti-OmpC appears here limited. Finally, if we combine results issued from the four possible tests (ASCA serum and/or ASCA supernatant and/or OmpC serum and/or OmpC supernatant) we reach the maximum sensitivity (70.2%). This improvement compared to ASCA alone sensitivity (serum or supernatant) is due to the fact that among the population with CD (n  =  67) we found 7 patients who were positive for anti-OmpC only. Despite these results combinations, 20 of 67 patients with CD (29.8%) were still negative for both tests (ASCA and anti-OmpC) in both matrices (serum and supernatant). The degree of agreement between the results of ASCA and anti-OmpC is poor (Kappa  =  0.033 in serum and Kappa  =  0.165 in the supernatant) which means that these two tests are not substitutable. Nevertheless, such a combination is expensive so, in our opinion, the contribution of anti-OmpC is negligible and does not justify its implementation in routine. Given the improvement of sensitivity of ASCA test by analyzing culture supernatant, we propose to use this test in immunological laboratories equipped for tissue cultures for helping diagnosis of CD.
